# Acute hospital admissions among nursing home residents: a population-based observational study

**DOI:** 10.1186/1472-6963-11-126

**Published:** 2011-05-26

**Authors:** Birgitte Graverholt, Trond Riise, Gro Jamtvedt, Anette H Ranhoff, Kjell Krüger, Monica W Nortvedt

**Affiliations:** 1 Centre for Evidence-Based Practice, Bergen University College, Post box 7030, N-5020 Bergen, Norway; 2 Department of Public Health and Primary Health Care, University of Bergen, Bergen, Norway; 3 Norwegian Knowledge Centre for the Health Services, Oslo, Norway; 4 Kavli's Research Centre for Ageing and Dementia, Haraldsplass Hospital, Bergen, Norway and Institute of Medicine, University of Bergen, Bergen, Norway; 5 Løvåsen Teaching Nursing Home, Municipality of Bergen, Bergen, Norway

**Keywords:** homes for the aged, nursing home, hospitalisation, patient admission

## Abstract

**Background:**

Nursing home residents are prone to acute illness due to their high age, underlying illnesses and immobility. We examined the incidence of acute hospital admissions among nursing home residents versus the age-matched community dwelling population in a geographically defined area during a two years period. The hospital stays of the nursing home population are described according to diagnosis, length of stay and mortality. Similar studies have previously not been reported in Scandinavia.

**Methods:**

The acute hospitalisations of the nursing home residents were identified through ambulance records. These were linked to hospital patient records for inclusion of demographics, diagnosis at discharge, length of stay and mortality. Incidence of hospitalisation was calculated based on patient-time at risk.

**Results:**

The annual hospital admission incidence was 0.62 admissions per person-year among the nursing home residents and 0.26 among the community dwellers. In the nursing home population we found that dominant diagnoses were respiratory diseases, falls-related and circulatory diseases, accounting for 55% of the cases. The median length of stay was 3 days (interquartile range = 4). The in-hospital mortality rate was 16% and 30 day mortality after discharge 30%.

**Conclusion:**

Acute hospital admission rate among nursing home residents was high in this Scandinavian setting. The pattern of diagnoses causing the admissions appears to be consistent with previous research. The in-hospital and 30 day mortality rates are high.

## Background

Elderly people often have several chronic conditions and co-morbidity that mostly require primary health care. Although the trend is to scale down institutional long-term care and develop home-based and assisted-living facilities, the frailest older people still need to be taken care of in skilled nursing homes [1].

Hospitalising older people places them at risk of experiencing what Creditor describes as a cascade of dependence [2]. More specifically, hospitalisation of nursing home residents has been found to often result in a decline in functional status and problems unrelated to the cause of admission [3,4]. Given the frailty of nursing home residents, the benefits of hospitalisation are often questioned. As such, reducing hospitalisation in the nursing home population (NHP) may potentially benefit not only these people but also health care systems that are currently trying to develop models that better coordinate care at lower levels [1,5]. Nursing home residents' usage of acute care services has been subject to investigation in a number of studies, representing different countries and health care systems from Australia [6-8], Canada [9-11], UK [12] and a range of US-based studies synthesised in a systematic review [13]. In a recent systematic review with particular focus on visits to Emergency Departments (EDs) an important finding, besides that transfers to EDs are common across nations, was that in at least 40% of the cases the nursing home resident is discharged back to the residential facility without admission [14]. This underscores the importance of distinguishing between visits to EDs and admissions to hospital when level of analysis is determined for different outcome measures. The systematic review restricted to US studies found that annual hospitalisation rates vary greatly between 9-59% [13]. The wide range was partly being attributed to different data sources and inconsistent definitions of what constitutes a hospitalisation.

We wanted to study acute hospital admissions in a nursing home population (NHP) in a Scandinavian setting, where the decentralised primary health care systems use substantially higher shares of gross domestic product (GDP) than the OECD average [15].

This study aimed to 1) determine the incidence of acute hospital admission among nursing home residents versus community dwellers and 2) to describe the hospital stays of the nursing home population according to diagnosis at discharge, length of stay and mortality.

## Methods

### Study population

**Figure 1:**
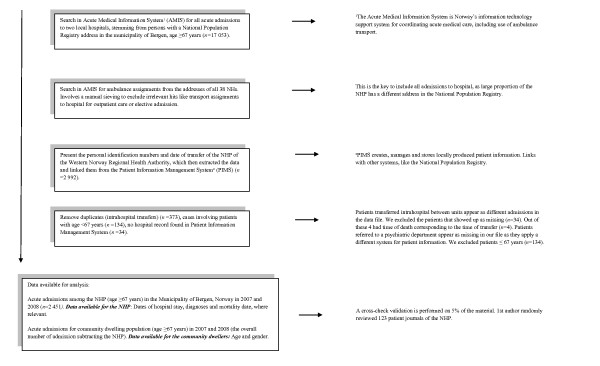
**Flow chart for data collection**.

We studied acute hospital admission rates among elderly people (age ≥ 67) in the Municipality of Bergen, Norway, a geographically well-defined area, from 1^st ^January 2007 to 31^st ^December 2008. The data were ascertained by retrospectively linking several databases with electronically coded data. We identified all cases through the Acute Medical Information System (AMIS) through the Bergen Hospital Trust under the Western Norway Regional Health Authority. There are two local hospitals in the health trust. The cases stemming from nursing homes were identified through ambulance records and investigated further. Specifically we investigated the hospital stays of the NHP for diagnosis at discharge, length of stay and mortality. This information was extracted electronically from hospital patient records corresponding to the personal identification number on the ambulance record and time of transfer (Figure 1). We did not include elective hospital admissions or admissions from lower levels of residential facilities, identified by defining the search by this criterion in AMIS.

Denominators for calculation of hospitalisation rates in the NHP were obtained from Statistics Norway, stratified by age and sex.

To validate the data material the first author did a cross-check of 123 patient journals, equal to 5% of the valid cases. The result of this was 100% accordance between the data file and the patient journals.

### Setting

The study was carried out in Bergen, an urban and suburban municipality in western Norway with a population of 247 746 (January 1^st ^2008; table 06913, Statistics Norway). Bergen had 38 nursing homes at the time of data collection with a total of 2 081 beds (range 17-174 beds per nursing home). The regulations governing nursing homes in Norway define them as residential care facilities that provide 24-hour health-related attention and care. The target population for NH beds is older people whose daily needs are not being met through skilled nursing care and ongoing chronic care in lower levels of residential care or through community-nursing. A requirement to apply for a long-term bed is that a lower level of services has been attempted prior to this. Further, short-term stays are mandatory to applicants of long-term beds and are used to assess and clear up the need for a long-term bed. Short-term nursing home beds are thus for elderly persons in the process of applying for a long-term bed or who are in a temporary need for the assistance offered here [16]. In Bergen, 16% of the residents aged ≥ 80 years live permanently in nursing homes. Norway's municipalities coordinate the decentralised responsibility for primary health care. Long-term care residents in Norway pay 85% of their monthly pension for residing in a nursing home. Each nursing home is assigned resources according to the number of residents and has financial autonomy. Among the 38 nursing homes, 18% of the beds are reserved for short-term stays. Most nursing homes combine long-term and short-term beds. Based on its geographical location, each nursing home is affiliated with one of two local hospitals, the same two that applies to all residents of this municipality. A peculiarity of this Norwegian setting is the fact that hospitalisations are equal to in-hospital admissions. Acute care is organised in a manner where referrals to hospital are made either from a community-based ED or a general practitioner.

### Characteristics of hospitalisation

The primary diagnosis at discharge, by the International Classification of Diseases version 10 (ICD-10), was retrieved through hospital-based patient records and presented in aggregated levels of the main chapters of the diagnosis system. Length of stay was calculated from admission and discharge dates. Date of death was ascertained by an existing link between the patient journal system and the National Population Registry. Based on this information, we present incidence of hospitalisation, diagnosis at discharge, length of stay, in-hospital mortality and 30 day mortality rates after discharge.

### Statistical methods

Incidences of hospitalisation were calculated based on person-time at risk during the two years study period. The overall number of individuals in nursing homes in each age and sex category during the two years was provided by Statistics Norway, for short- and long term stays respectively. To estimate the person-time at risk of being hospitalised for the NHP we initially estimated the mean duration of short- and long term stays by dividing the overall bed-years for short- and long term beds respectively, by the number of residents in each type of bed during one year. These estimates of duration of stays are the equivalent to the time at risk of being hospitalised for each type of stay. These estimates were then multiplied by the number of individuals in each age and sex category for the two different types of stays each year to give the total person-time at risk for each age and sex category for the NHP. The person-time at risk for the community dwellers was calculated by subtracting the person-time at risk for the NH population from the person-time at risk for total population (≥ 67 years). The latter was simply calculated as the sum of individuals in each age and sex category in the general population January 1^st ^2007 and January 1^st ^2009 (Statistics Norway). The hospital admission incidence was calculated by dividing the number of admissions by the estimated number of person-years in both populations, stratified by age and sex (table 1). This enabled us to calculate rate ratios for hospital admission for the NHP and the community dwellers.

**Table 1:** Rates of acute hospital admissions in a nursing home population and the corresponding community dwelling population

	Nursing home population 2007-2008									Community-dwelling population 2007-2008*								
	
	Total			Men			Women			Total			Men			Women		
	
	Person-years	Admissions (*n*)	Mean annual admission rate	Person-years	Admissions (*n*)	Mean annual admission rate	Person-years	Admissions (*n*)	Mean annual admission rate	Person-years	Admissions (*n*)**	Mean annual admission rate	Person-years	Admissions (*n*)	Mean annual admission rate	Person-years	Admissions (*n*)	Mean annual admission rate
**Total**	3 927	2 451	0.6	1 129	930	0.8	2 798	1 521	0.5	58 196	15 052	0.3	23 781	6 574	0.3	37 230	8 478	0.2

**67-79 years**	740	531	0.7	316	274	0.9	424	257	0.6	39 296	6 767	0.2	17 268	3 471	0.2	21 712	3 296	0.2

**80-89 years**	1 941	1 322	0.7	586	493	0.8	1 354	829	0.6	16 690	6 787	0.4	5 995	2 685	0.5	10 110	4 102	0.4

**90+ years**	1 246	598	0.5	227	163	0.7	1 019	435	0.4	2 210	1 498	0.7	518	418	0.8	1 465	1 080	0.7

**Notes**: *The number of person-years for the community-dwelling population is based on the mean numbers from Table 05277 from Statistics Norway (2007 and 2009).

**The overall number of admissions minus the admissions from the nursing home population.

The confidence intervals are based on Poisson distribution.

The NHP was further analysed for demographics, length of stay and mortality according to the primary diagnosis at discharge, employing main chapters of the ICD-10. In addition to in-hospital mortality, we studied 30 day mortality after discharge, using the first admission if more than one during the study period.

Age was presented in three categories from the data provider. The estimated mean age is based on the median value in each age category weighted by the number of cases in each category.

We used SPSS version 17 for all analysis.

### Ethics

This study was approved by the The Regional Committee for Medical and Health Research Ethics, Western Norway, and The Privacy Ombudsman for Research, Norwegian Social Science Data Services (NSD). The Privacy Ombudsman for Research has exempted the project from obtaining patient consent forms.

## Results

In the municipality of Bergen we found a total of 17 053 emergency hospital admissions during a two-year study period in a population of 31 162 persons ≥ 67 years (January 1^st ^2008, Statistics Norway, table 06087). Of these, there were 2 451 cases of ambulances transporting nursing home residents to hospital. These represent the hospital admissions of the NHP, which we investigated further: The 2 451 admissions constituted 1 668 individual people with an estimated mean age of 85 years, and 38% were men. There were 1 184 cases (879 people) in 2007 and 1 267 cases (903 people) in 2008. Out of the total number of hospitalisations stemming from the NHP, 27% represent people with multiple admissions during the study period.

### Incidence of acute hospital admission - both populations

The mean admission rate was 0.62 admissions per person-year (95% confidence interval (CI): 0.60-0.65) for the nursing home residents versus 0.26 (95% CI: 0.25-0.26) for the community dwellers (Table 1). The admission rate increased with ascending age among the community dwellers and descending age among the nursing home residents. In fact, for the age category 90+ years, the community dwellers had a higher admission incidence than that of the nursing home residents. Men had higher admission rates than women in all age categories in both populations. Interestingly, this sex difference was markedly larger within the NHP.

### Diagnoses at discharge - nursing home population

The most common main diagnoses at discharge (Table 2) were diseases of the respiratory system (ICD-10 J00-J99), responsible for 20% of the admissions among the NHP. The most common diagnosis within this system was pneumonia (J13-J18.9), counting for 71% (*n *= 344). Pneumonia as a single diagnosis accounted for 14% in the studied NHP.

**Table 2:** Distribution of primary diagnoses at discharge in the nursing home population. Sex, length of stay and mortality according to primary diagnosis

ICD-10 codes - main chapter	Primary diagnosis at discharge *n *(%)	Men *n *(%)	Median length of stay (Inter quartile range)	Mortality in hospital *n *(%)	Mortality ≤ 30 days after discharge *n *(%)
Certain infectious and parasitic diseases (A00-B99)	173 (7.1)	78 (45.1)	5 (6)	44 (25.8)	64 (37.0)

Neoplasms (C00-D48)	94 (3.8)	38 (40.4)	4 (7)	20 (21.2)	47 (50.0)

Diseases of the circulatory system (I00-I99)	405 (16.5)	141 (34.8)	3 (4)	94 (22.2)	139 (34.3)

Diseases of the respiratory system (J00-J99)	486 (19.8)	217 (44.6)	4 (6)	137 (28.1)	208 (42.8)

Diseases of the digestive system (K00-K93)	243 (9.9)	90 (37.0)	3 (4)	28 (11.5)	61 (25.1)

Diseases of the genitourinary system (N00-N99)	162 (6.6)	83 (51.2)	3 (4)	19 (11.7)	38 (23.5)

Symptoms, signs and abnormal clinical and laboratory findings, not elsewhere classified (R00-R99)	140 (5.7)	66 (47.1)	1 (1)	15 (10.7)	27 (19.3)

Injury, poisoning and certain other consequences of external causes (S00-T98)	437 (17.8)	113 (25.9)	3 (4)	18 (4.1)	63 (14.4)

Other ICD-10 codes	311 (12.7)	104 (33.4)	-	29 (9.3)	60 (19.2)

**Total**	**2 451 (100)**	**930 (37.9)**	**3 (4)**	**404 (16.4)**	**707 (28.8)**

Diagnoses related to injury, poisoning and certain other consequences of external causes (S00-T98) were associated with 18% of overall admissions (*n *= 437). Based on a discretionary reading of the single diagnoses, 82% (*n *= 358) of these related to falls (S010-S934). Fall-related admissions counted for 15% of overall admissions from the NHP. Confirmed diagnoses of fractures of the femur accounted for 10% of overall admissions (*n *= 247).

Diseases of the circulatory system (I00-I99) accounted for 17% of the primary diagnoses. In this domain, ischaemic heart disease (I20-I25) comprised 24% (*n *= 98) and cerebrovascular disease (I60-I69) 24% (*n *= 96) of the primary diagnoses.

On average, the hospitalised nursing home residents had 3.4 secondary diagnoses (range 0-9), and 69% had 4-7 secondary diagnoses.

### Length of stay - nursing home population

The overall median length of stay for the NHP was 3 days (mean = 5.1, SD ± 6.1, range 1-73, interquartile range (IQR) = 4). The longest hospital stays were related to infectious diseases (median = 5, IQR = 6), neoplasms (median = 4, IQR = 7), respiratory diseases (median = 4, IQR = 6) and circulatory diseases (median = 3, IQR = 4).

### Mortality - nursing home population

There was a 16% in-hospital mortality rate in the NHP. The overall mortality rate 30 days after discharge increased to 29% (Table 2). In-hospital mortality was highest for respiratory diseases (30%), infectious diseases (24%), neoplasms (23%) and circulatory diseases (23%) (Table 2). Within 30 days of discharge, 43% of the patients admitted with respiratory diseases had died. Other disease domains that had a high mortality rate at 30 days were neoplasms (49%), infectious diseases (35%), circulatory diseases (34%) and digestive diseases (25%).

The diagnoses associated with the lowest in-hospital mortality and 30 day mortality were injury, poisoning and other external causes: 4% in-hospital mortality and 14% 30 day mortality rate after discharge.

## Discussion

Nursing home residents had a rate of 0.62 hospital admissions per person-year, more than twice as high as the community dwellers (0.26). When investigating the hospital admissions of the NHP further, we found the top three causes of admission were respiratory diseases, injury, poisoning and other external causes and circulatory diseases, accounting for 55% of the admissions. The overall median length of hospital stay was 3 days (IQR = 4); the in-hospital mortality rate was 16% and 30 day mortality rate after discharge 29%.

### Methodological issues

There are a large number of studies that have examined nursing home residents' usage of acute hospital services, but several methodological differences reduce the comparability of the findings. First of all this relates to whether hospitalisation rates are defined as ED visits or in-hospital admissions. The current study focuses solely on the latter as referrals to hospital equalises hospital admission in this setting. Arendts & Howard [14] found that at least 40% of visits to ED are returned to the nursing home without admission. Conceptually, the principles of our setting versus other settings are perhaps not that different; instead of the initial assessment of the patient in the hospital-based ED, this assessment is done in a community-based ED facility. However, juxtaposing these conceptual frameworks and assuming they essentially are the same, may be a mistake and instead play a confounding impact on the high rates we found. If there is a lower threshold for referring to hospital in a community-based ED than a hospital-based ED, this may support such a supposition. On the other hand, though, the same principles apply for the remaining population and this should have resulted in equally high rates for them. Instead we found rate ratios indicative of the opposite; the NHP is hospitalised disproportionally more often than their community dwelling peers, compared to other studies reporting on this [8,11,12].

A second issue complicating comparability is the different levels of care applying to nursing homes in different settings. The present study is only investigating hospital admissions from nursing homes offering the highest level of residential care in Norway, a level referred to as skilled nursing homes in the literature. Two effects could result by this; for one it is known that lower levels of care have higher incidence of hospitalisation than higher level facilities [13] and secondly this may affect the distribution of diagnoses, due to resident-level differences as well as level of care offered in the different settings. Thirdly, observational periods in published studies vary widely [13,14] and many of them may be considered rather old.

### Annual rates of hospitalisation

The annual hospital admission rate we found was distinctly higher than in previous population-based studies of nursing home residents with observational periods similar to ours. Annual rates of 16-26% were found in the United Kingdom [12] and North America [9-11,17], markedly lower than our incidence of 62%. Further, the rate ratios between nursing home residents and community dwellers deviate substantially from ours and reinforce our claim that annual incidence rates are high for this Norwegian NHP. Others report rate ratios between nursing home residents and community dwellers of 1.01-1.69, whereas our study suggests an equivalent ratio of 2.3 [8,11,12]. Plausible explanations for this may be different conceptions of what a nursing home is and what level of care should be offered in this setting. These divergences may reflect a lower level of both acute care and palliative care in the Norwegian nursing home setting, an assertion supported by the high annual admission rate and the high in-hospital mortality rate. As only the frailest and the sickest of the elderly will achieve a nursing home bed in this setting, another explanation could be that the studied NHP are frailer than other NHPs that have undergone investigation, resulting in high annual hospitalisation rate.

Male nursing home residents had far more hospital admissions than their community-dwelling peers of similar age. Male sex and increasing age are recognised to predispose towards hospitalisation, and this coincides with our findings of higher annual admission rates among men than among women in both populations [18]. However, this disproportion between sexes was much stronger among the nursing home residents (0.82/0.54 = 1.52) than among the community dwellers (0.28/0.23 = 1.22), a finding that remains to be interpreted. Further, age consistently follows a pattern that is reversed for the two populations: nursing home residents gradually have fewer hospital admissions as they age, whereas admissions among community dwellers increased markedly with increasing age. This negative correlation between age and hospitalisation in the NHP reflects that older nursing home residents are withheld in the nursing home in case of acute incidences, and probably that they are more likely to die in the nursing home without being hospitalised. Moreover, the increase in admissions among the community dwellers may indicate that hospital becomes a social safety net for frail older community dwellers.

### Characteristics of hospital stays - nursing home population

The combinations of diagnoses dominant in our material correspond to findings in comparable studies [8,10-12,17]. As such, strategies aiming at governing cardiovascular, respiratory and falls-related conditions should be given priority, as they can potentially benefit the largest part of patients across countries and models of aged care.

Length of stay is a common outcome when investigating hospitalisations of nursing home residents. We found a median length of stay of 3 days (IQR = 4), less than other studies, ranging from 6 to 14 days [4,8]. The significance of the different findings of this is not clear; however, it does raise the question as to why practice is heterogeneous. In any case, this finding is a reflection of a pattern of care that indicates a low threshold for hospitalising and a corresponding low threshold for return to the nursing home. This finding may be indicative of an underpinning, implicit policy that differs between Norway and other countries that have been studied.

The decision to hospitalise nursing home residents is a complex issue and relies on multiple factors attributable to the individual resident, the nursing home facility and policy incentives [19]. As such, this study only investigated a fragment of a complex framework across different levels of health care. Nevertheless, this study contributes to a field of practice previously undescribed in Norway and comparable, neighbouring countries. Such basic health statistics are necessary to pinpoint further studies in this field, in addition to being a guide for setting priorities and health policy decisions.

The literature presents several promising interventions aimed at reducing the number of hospital admissions of people in long-term care settings [20,21]. Although some of the models identified would benefit from further research, they offer optimism for reducing hospital admissions among frail old nursing home residents and point to different strategies for doing so. Further, suggestive frameworks are increasingly applied to decide which conditions are sensitive to ambulatory care and may be prevented from hospitalisation by intervention in primary care [22-24]. Analysing the data in this investigation according to such a perspective would be useful.

### Limitations and strengths of this study

A shortcoming of this study is the lack of possibility to stratify cases of hospitalisation by resident status. Short-term residents are shown by the literature to be hospitalised more often than long-term residents and may in addition affect the distribution of diagnoses. Still, a two-year observational period with population-based data does provide a reliable reflection of our study objectives. Another limitation, also linked to the impediments in the system for retrieving relevant data across levels of primary and secondary care, is the missed opportunity to link the hospital-based data to nursing home-based data. This would have enabled assessment for outcomes of hospitalisation other than mortality.

Electronically linked data are generally viewed as a limitation. However, we manually reviewed of a subsample of 5% of cases to validate data quality and findings and found 100% accordance.

## Conclusions

We studied acute hospital admissions rates among elderly people over two years in a well-defined Norwegian population. Our findings support previous literature that transfers to hospital for acute care is common among nursing home residents. Our annual incidence was 0.62, which is even higher than reported previous in population-based studies and 2.3 times higher than among the corresponding community dwellers. We found that respiratory, cardiovascular and falls-related diagnoses were most common and accounting for more than half of the diagnoses, in line with the pattern found in previous studies. Additionally we found higher in-hospital and 30 days after discharge mortality rates than previously reported, but a shorter length of stay. The substantial variation in hospitalisation and mortality rates between studies may indicate that there are different thresholds for transferring nursing home residents to hospital in case of acute flares, across different settings. Further, features of welfare model and organisation of health care services specific to Scandinavia may have impacted on the results of this study. Nevertheless, the results point to the need for increased attention towards the supply of services for acute and palliative care in the nursing homes.

## Competing interests

The authors declare that they have no competing interests.

## Authors' contributions

**KK **made substantial contribution to conception and design of the study, as well as critically revising the manuscript. **AHR and GJ contributed to the process **of analysis and interpretation of findings and critically revising the manuscript. **TR**, **MWN **and **BG **contributed to the design of the study, the acquisition, interpretation and analysis of the data, as well as drafting and critically revising the manuscript.

**All authors **read and approved the final manuscript.

## Pre-publication history

The pre-publication history for this paper can be accessed here:

http://www.biomedcentral.com/1472-6963/11/126/prepub
